# Why stillbirth deserves a place on the medical school curriculum: Stillbirth‐related teaching in UK medical schools

**DOI:** 10.1111/1471-0528.15939

**Published:** 2019-10-07

**Authors:** Krithi Ravi, Shannon Gunawardana, Krupa Ravi, Movin Abeywickrema, Monica Davies, Emily Tough, Maureen Kelley

**Affiliations:** ^1^ Medical Sciences Division University of Oxford Oxford UK; ^2^ Ethox Centre Nuffield Department of Population Health Big Data Institute University of Oxford Oxford UK; ^3^ Wellcome Centre for Ethics and Humanities Nuffield Department of Population Health Big Data Institute University of Oxford Oxford UK

The arguments included in this article are outlined in greater detail online in an accompanying full‐length discussion (see Appendix S1).

Care received by parents following a stillbirth has a substantial impact on their grieving process (Nuzum et al. *PLoS One* 2018;13:e0191635). It is crucial to have healthcare workers who can offer appropriate, compassionate care to parents mourning a stillbirth. This includes not just the named consultant and midwife but all members of the healthcare team, including medical students. Our survey of 35 UK state medical schools (40% response rate) showed that only 57% covered stillbirth somewhere in their curriculum. We believe incorporating stillbirth into medical school curricula would improve care for bereaved parents by allowing medical students to thoughtfully engage in their care, while equipping future obstetricians with the empathy and interpersonal skills required to manage this devastating event.

Unfamiliarity with stillbirth owing to lack of formal teaching increases the risk of substandard care for bereaved parents at a stillbirth, which is especially significant given that behaviours of staff have been shown to have a memorable long‐term impact on parents (Ellis et al. *BMC Pregnancy Childbirth* 2016;16:16). Experienced obstetric consultants have often conveyed that they ‘learned on the job’ and had not previously ‘received any specialist training in perinatal bereavement care’ (Nuzum et al. *Arch Dis Child Fetal Neonatal Ed* 2013;98:A90). Generic communication skills training on ‘breaking bad news’ often proves insufficient at stillbirths, because they occur at a time when parents are looking to welcome a new beloved member into their family. This element of shock is exacerbated by a busy labour ward environment in which other children are being born. Consequently, healthcare staff and students hesitate to express their sympathy to parents or even acknowledge the passing of their child. The absence of acknowledgement of the loss of a child – ‘the people who said nothing’ – can have a devastating impact on parents (Kelley and Trinidad *BMC Pregnancy Childbirth* 2012;12:137). Furthermore, this silence around stillbirth reinforces the social stigma that makes it a hidden grief for many parents.

Following a stillbirth, care for the parents can be somewhat neglected amidst the focus on neonatal resuscitation and meticulous documentation. The medical student's role in a stillbirth is unique in that they do not have pressing medico‐legal and clinical duties to attend to, allowing them to assist in supporting bereaved parents, and highlight parents’ needs to other members of the team. However, owing to the paucity of stillbirth teaching at medical school, students may be actively excluded from patient contact at a stillbirth owing to concerns about their inexperience and ability to comport themselves sensitively.

Within the already crowded medical curriculum, it can be very challenging to introduce new topics but stillbirth teaching could fit closely with existing practical education on communication and bereavement during clinical placements, rather than replacing other didactic teaching. Medical school teaching can be a powerful tool to combat the taboo surrounding stillbirth, as was the case with mental health and palliative care. Talking openly about stillbirth, from both a clinician and patient perspective, allows students to feel more comfortable discussing it with their peers, healthcare staff and bereaved parents. Allowing medical students to engage emotionally with patient tutors, and to practice communicating effectively in role‐play scenarios, will generate empathetic clinicians and compassionate future obstetricians.

## Disclosure of interests

None declared. Completed disclosure of interests form available to view online as supporting information.

## Contribution to authorship

Krithi R, SG, Krupa R, MA, MD and ET conceived the idea for the Commentary and performed the literature review. Krithi R, SG, MA, MD and ET contacted medical schools for the survey. Krithi R established that the survey was exempt from ethical approval, took the lead in writing the online manuscript and wrote the final version of the published manuscript. SG contributed the personal reflection included in the online manuscript. Krupa R wrote the final version of the online manuscript. MK supervised the survey and the writing of the manuscript. All authors provided critical feedback that helped to shape the manuscript.

## Details of ethics approval

The survey of UK medical schools was deemed to fall outside the remit of the Central University Research Ethics Committee at the University of Oxford and was therefore exempt from ethics approval.

## Funding

The authors have not received any funding for this article. Maureen Kelley’s efforts were supported by the Wellcome Trust Strategic Award (Grant No. 096527) and the Wellcome Trust and MRC Newton Fund Collaborative Award (Grant No. 200344/Z/15/Z).

## Supporting information


**Appendix S1**. Expanded discussion, ‘Why stillbirth deserves a place on the medical school curriculum’.
