# Supplementary material for: Why stillbirth deserves a place on the medical school curriculum: Stillbirth‐related teaching in UK medical schools
Source: BJOG. 2019 Oct 7;127(3):414. doi: 10.1111/1471-0528.15939 (PMC7004000; doi:10.1111/1471-0528.15939)
Supplement: Supplementary file 1 — Appendix S1. Expanded discussion, ‘Why stillbirth deserves a place on the medical school curriculum’. [file BJO-127-414-s001.pdf]

Why stillbirth deserves a place on the medical school curriculum: Commentary

Authors: Krithi Ravi[1], Shannon Gunawardana[1], Krupa Ravi[1], Movin Abeywickrema[1], Monica Davies[1], Emily Tough[1], Maureen Kelley[2, 3]

1. Medical Sciences Division, University of Oxford

   Level 2, Academic Block

   John Radcliffe Hospital

   Oxford

   OX3 9DU

2. Ethox Centre

   Nuffield Department of Population Health, University of Oxford

   Big Data Institute

   Old Road Campus

   Oxford

   OX3 7LF

3. Wellcome Centre for Ethics and Humanities

   Nuffield Department of Population Health, University of Oxford

   Big Data Institute

   Old Road Campus

   Oxford

   OX3 7LF

Corresponding author: Krithi Ravi

- Address: 60, Durley Avenue, Pinner, Middlesex, HA5 1JH
- Email address: kkravi7@gmail.com
- Telephone number: +44 7890232100

Running title: Stillbirth-related teaching in UK medical schools

Main body of text:

As a medical student on my O&G rotation, I met a couple who were expecting their first child, a girl. When she was born, one of the doctors cut the cord and explained to the parents that the baby had to be taken to the resuscitaire as she was 'a bit sleepy'. The room grew noisy with voices, which were cut through by the baby's silence. My view was obscured by the numerous midwives and doctors around the baby but I stayed with Mum and Dad along with their midwife and tried my best to comfort them. Numerous teams entered and left the room. After some time, the doctor informed the parents that it would be best to stop resuscitation attempts. They were offered the opportunity to hold their baby. I wanted to stay with Mum and Dad to offer comfort but I was very upset and thought it inappropriate for my grief to cloud their mourning, so I left the room. The next day, I tried to find the parents to offer my condolences but they had left hospital soon after their baby was sent for post mortem. I regret not looking for them earlier in the day. I have never witnessed grief as I saw in that room and wish there was more I could have done to help Mum and Dad.

INTRODUCTION

Care received by parents following a stillbirth has a substantial impact on their grieving process[1]. Therefore, it is crucial to have healthcare workers who can offer appropriate, compassionate care to parents grieving a stillbirth. This includes not just the named consultant and midwife, but 'all professionals likely to be involved'[2], including medical students. Currently, stillbirth does not feature in a significant proportion of medical school curricula. We performed an e-mail and telephone survey of 35 UK state medical schools, asking 1) if they covered stillbirth in their curriculum and 2) when and how stillbirth-related teaching was delivered. We received responses from 14 (40%) medical schools, of which 8 (57%) mentioned stillbirth somewhere in their curriculum, 5 (36%) covered communication skills in the circumstance of a stillbirth, and 2 (14%) used patient-led teaching.

The lack of stillbirth teaching in medical schools is understandable given the relatively low incidence of stillbirth in the UK compared to global incidence[3]. However, the rate of stillbirths in the UK remains high compared to rates in similar high-income countries, and has stayed static since 2015[3].

We argue that incorporating stillbirth into medical school curricula would improve care for bereaved parents by allowing medical students to thoughtfully engage in their care, whilst equipping future obstetricians with the empathy and interpersonal skills required to manage this devastating event.

THE COST OF INADEQUATE STILLBIRTH TEACHING AT MEDICAL SCHOOL

Stillbirth can be a chaotic experience for parents due to the rapid arrival of resuscitation teams and senior figures. Amidst the focus on neonatal resuscitation and meticulous documentation, care for the parents can be somewhat neglected. The medical student's role in a stillbirth is unique in that they do not have pressing medicolegal and clinical duties to attend to, allowing them to support bereaved parents. This is illustrated by a stillbirth narrative in which a medical student 'detaches herself from the group and… gently takes the woman's hand.' - this action is met with 'silent gratitude' and contrasts with the inattention from the rest of the clinical team[4].

Unfortunately, current medical school teaching inadequately equips medical students to support grieving parents during a stillbirth. Furthermore, students may be actively excluded by healthcare professionals from events such as a stillbirth due to concerns about their ability to comport themselves sensitively and the emotional ramifications for students.

In not being exposed to stillbirth-related teaching at medical school we also perpetuate a vicious cycle in which lack of exposure leads to a lack of experience in dealing with distressing situations. This unfamiliarity increases the risk of substandard care for bereaved parents at a stillbirth, which is especially significant given that behaviours of staff have been shown to have a memorable long-term impact on parents[5]. We should aim to prevent situations in which a qualified doctor looking after grieving parents feels unprepared and professionally isolated, inadvertently reinforcing the social stigma surrounding stillbirth that makes it a hidden grief for many. Stillbirths occur on a continuum of perinatal to neonatal deaths, and exposure to bereavement skills at each stage is an essential part of a compassionate medical education.

Experienced obstetric consultants have often conveyed that they ‘learned on the job’ and had not previously ‘received any specialist training in perinatal bereavement care’[6]. This mirrors the concern expressed by midwifery students, who reported that they were ‘not prepared for it’ and didn’t ‘know what to say’[7]. A heartfelt account in a study by Nuzum *et al.* captures the isolation felt by a junior clinician following a stillbirth: ‘“There was no recognition that it might be difficult...there was no training... You did it yourself … nobody cared if you got so psychiatrically disturbed you threw yourself off the roof the following week.”’[8].

Finally, difficulty coping with uncertainty at the time of stillbirth appears to be a prominent feature of both parents' and healthcare professionals’ experiences, sometimes leading to an unfortunate breakdown of the doctor-patient relationship. The uncertainty surrounding the cause of the stillbirth can lead to healthcare professionals avoiding bereaved parents. For example, student midwives reported that they ‘felt awkward being with a bereaved women unless they were engaged in some activity’[7]. There is a risk that some parents may react with distrust, perceiving clinicians’ silence as defensiveness[9]: a multi-centre case study on care after stillbirth found that parents did not like being left on their own for long periods of time without information[2]. Thus, there is a need for teaching around managing uncertainty whilst delivering compassionate care in the event of stillbirth.

THE BENEFITS OF STILLBIRTH TEACHING AT MEDICAL SCHOOL

The obvious benefit of including stillbirth teaching in the medical school curriculum is to prepare future obstetricians to effectively care for parents at a stillbirth. However, we would like to highlight a few immediate benefits to medical students from being taught about stillbirth at medical school.

It could be argued that teaching of ‘breaking bad news’ communication skills is sufficient to equip medical students to handle the emotional burden of bereavement at a stillbirth. However, stillbirth exposes students to death in a different context to that in which it is usually encountered. It comes at a

time when parents are looking to welcome a new beloved member into their family after months of preparation. This element of shock is exacerbated by a busy labour ward environment in which other children are being born. Consequently, healthcare staff and students hesitate to express their sympathy to parents or even acknowledge the passing of their child. The absence of acknowledgement of the loss of a child - 'the people who said nothing' - has a negative impact on parents[9]. Mothers felt that healthcare professionals were 'uncertain, avoidant and tenuous' when referring to the stillbirth[10]. Critically, parents have repeatedly expressed that they appreciate emotional engagement and empathy from doctors[9]. In one study, parents reported being 'very touched by the moist eyes and apparent upset shown by professionals of all types'[11]; another parent felt that '[their doctor] was very compassionate' upon hearing that he had cried after the stillbirth[9]. Moreover, parents valued healthcare staff 'who overtly acknowledged their baby's existence as a much loved child'[11]. Medical students are in a unique position to offer empathy and validation, support that is not time-limited, and could even highlight parents' needs to busier members of staff.

Finally, stillbirth teaching at medical school can be a powerful way of combating the taboo surrounding stillbirth. In addition to the devastation of losing their child, parents suffer the effects of stillbirth-related stigma. They are 'not able to grieve openly...to openly celebrate or remember their baby's birth or death'[9]; they feel 'isolated by the lack of support and understanding that they receive'[5]; they feel that 'mourning the death of a newborn [is] taboo and not culturally acceptable'[12]. Mental health and palliative care are examples of topics which have been at least partially destigmatised and incorporated into mainstream healthcare through medical education. Similarly, talking openly about stillbirth, from both a clinician and patient perspective, during medical school allows students to feel more comfortable discussing it with their peers, healthcare staff and bereaved parents.

HOW COULD STILLBIRTH TEACHING BE IMPLEMENTED?

Stillbirth-related teaching need not come at the cost of lower quality patient care - we are not advocating that inexperienced students practise their skills with vulnerable patients. In a study of

consultant obstetricians, many explained that it is 'more your expression' and 'your body language' that makes the difference for the patient[6]. Therefore, teaching could be provided in small group settings using patient tutors and role-play, as is the case for many other communication skills taught in medical school. Allowing medical students to engage emotionally with patient tutors sharing their experiences of stillbirth, and to practice communicating effectively in role-play scenarios provides a framework which can be built on during specialist training. Specific case-based discussions can be used to explore the management of clinical uncertainty at a stillbirth. Inspiration can be found from other aspects of the medical school syllabus which refer to the communication of uncertainty to patients, such as prognosis in terminal illnesses.

Students should also be taught how stillbirth is an area where delivery of care rather than medicalisation is the means of therapy**.** Here, compassionate care requires switching from 'ritualising guidelines'[5] to clear communication, as well as provision of coping strategies and bereavement spaces[2]. Doctors need to act as emotional pillars for patients, facilitating their expression of grief. However, they often struggle with the 'shift from physician to grief counselor'[9] due to their additional clinical responsibilities. There is a need for this training especially given deficits in specialised staff, such as bereavement midwives[11]. Emotional support techniques such as taking the time to 'be present with (the parents) in their sadness'[9] and validating parents' experiences of grief need to be highlighted as valid management strategies that medical students and clinicians can use.

CONCLUSION

Incorporating stillbirth teaching into medical school curricula has the potential to improve perinatal bereavement care by enabling medical students to provide emotional support for grieving parents. In addition, structured teaching addresses the stillbirth-related stigma, thereby ameliorating the isolation experienced by both parents and clinicians. Specifically, medical schools could facilitate opportunities to learn from patient tutors and practise providing compassionate care to grieving parents in small group settings. Ultimately, this teaching will generate obstetricians who are confident in managing

and coping with a stillbirth. More immediately, this training would equip students to be mindful of parents’ needs and sensitively support them during this distressing event.

Disclosure of Interests: The authors declare no conflicts of interest.

Contribution to Authorship:

Krithi R., S.G., M.D., Krupa R., M.A. and E.T. conceived of the idea for the Commentary and performed the literature review. Krithi R., S.G., M.D., M.A. and E.T. contacted medical schools for the survey. Krithi R. established that the survey was exempt from ethical approval and took the lead in writing the manuscript. S.G. contributed the personal reflection included in the manuscript. Krupa R. wrote the final version of the manuscript. M.K. supervised the survey and the writing of the manuscript. All authors provided critical feedback which helped to shape the manuscript.

Details of Ethical Approval:

The survey of UK medical schools was deemed to fall outside the remit of the Central University Research Ethics Committee at the University of Oxford and was therefore exempt from ethics approval.

References

1. Nuzum D, Meaney S, O'Donoghue K. The impact of stillbirth on bereaved parents: A qualitative study. PLoS One [Internet]. 2018 [cited 2019 Jul 18];13(1):e0191635. Available from: http://www.ncbi.nlm.nih.gov/pubmed/29364937
2. Siassakos D, Jackson S, Gleeson K, Chebsey C, Ellis A, Storey C. All bereaved parents are entitled to good care after stillbirth: a mixed-methods multicentre study (INSIGHT). BJOG An Int J Obstet Gynaecol [Internet]. 2018 Jan 1 [cited 2019 Jul 18];125(2):160–70. Available from: http://doi.wiley.com/10.1111/1471-0528.14765
3. MBRRACE-UK Perinatal Mortality Surveillance Report [Internet]. 2018 [cited 2019 Jul 18]. Available from: www.hqip.org.uk/national-programmes.
4. de Zulueta PC. Suffering, compassion and 'doing good medical ethics.' J Med Ethics [Internet]. 2015 Jan 16 [cited 2019 Jul 18];41(1):87–90. Available from: http://www.ncbi.nlm.nih.gov/pubmed/25516944
5. Ellis A, Chebsey C, Storey C, Bradley S, Jackson S, Flenady V, et al. Systematic review to understand and improve care after stillbirth: a review of parents' and healthcare professionals' experiences. BMC Pregnancy Childbirth [Internet]. 2016 Jan 25 [cited 2019 Jul 18];16:16. Available from: http://www.ncbi.nlm.nih.gov/pubmed/26810220
6. Nuzum D, Meaney S, O'Donoghue K. PP.29 The Personal and Professional Impact of Stillbirth on Consultant Obstetricians. Arch Dis Child - Fetal Neonatal Ed [Internet]. 2013 Apr 26 [cited 2019 Jul 18];98(Suppl 1):A90.1-A90. Available from: http://fn.bmj.com/lookup/doi/10.1136/archdischild-2013-303966.309
7. Alghamdi R, Jarrett P. Experiences of student midwives in the care of women with perinatal loss: A qualitative descriptive study. Br J Midwifery [Internet]. 2016 Oct 2 [cited 2019 Jul 18];24(10):715–22. Available from: http://www.magonlinelibrary.com/doi/10.12968/bjom.2016.24.10.715
8. Nuzum D, Meaney S, O'Donoghue K. The impact of stillbirth on consultant obstetrician gynaecologists: a qualitative study. BJOG An Int J Obstet Gynaecol [Internet]. 2014 Jul 1 [cited 2019 Jul 18];121(8):1020–8. Available from: http://doi.wiley.com/10.1111/1471-

0528.12695

9. Kelley MC, Trinidad SB. Silent loss and the clinical encounter: Parents' and physicians' experiences of stillbirth–a qualitative analysis. BMC Pregnancy Childbirth [Internet]. 2012 Dec 27 [cited 2019 Jul 18];12(1):137. Available from: https://bmcpregnancychildbirth.biomedcentral.com/articles/10.1186/1471-2393-12-137

10. Nordlund E, Börjesson A, Cacciatore J, Pappas C, Randers I, Rådestad I. When a baby dies: Motherhood, psychosocial care and negative affect. Br J Midwifery [Internet]. 2012 Nov 16 [cited 2019 Jul 18];20(11):780–4. Available from: http://www.magonlinelibrary.com/doi/10.12968/bjom.2012.20.11.780

11. Downe S, Schmidt E, Kingdon C, Heazell AEP. Bereaved parents' experience of stillbirth in UK hospitals: a qualitative interview study. BMJ Open [Internet]. 2013 Jan 1 [cited 2019 Jul 18];3(2):e002237. Available from: http://www.ncbi.nlm.nih.gov/pubmed/23418300

12. Burden C, Bradley S, Storey C, Ellis A, Heazell AEP, Downe S, et al. From grief, guilt pain and stigma to hope and pride – a systematic review and meta-analysis of mixed-method research of the psychosocial impact of stillbirth. BMC Pregnancy Childbirth [Internet]. 2016 Dec 19 [cited 2019 Jul 18];16(1):9. Available from: http://www.ncbi.nlm.nih.gov/pubmed/26785915
